# Investigating Unhealthy Behaviors Associated with SF-36 Domains in Women with Endometriosis—Findings from a Web-Based Survey Data Set

**DOI:** 10.3390/bs14030199

**Published:** 2024-03-01

**Authors:** Mario Muselli, Martina Mancinelli, Erika Limoncin, Chiara Lolli, Erika Pelaccia, Maurizio Guido, Leila Fabiani, Stefano Necozione, Vincenza Cofini

**Affiliations:** 1 Department of Life, Health and Environmental Sciences, Public Health Section, University of L’Aquila, 67100 L’Aquila, Italy; martina.mancinelli@student.univaq.it (M.M.); erika.pelaccia@graduate.univaq.it (E.P.); maurizio.guido@univaq.it (M.G.); leila.fabiani@univaq.it (L.F.); stefano.necozione@univaq.it (S.N.); vincenza.cofini@univaq.it (V.C.); 2 Department of Dynamic, Clinical Psychology and Health Sciences, Sapienza University of Rome, 00185 Rome, Italy; erika.limoncin@uniroma1.it; 3 Department of Molecular Medicine, Sapienza University of Rome, 00185 Rome, Italy; chiara.lolli@uniroma1.it

**Keywords:** health-related quality of life, SF-36, endometriosis, unhealthy behaviors, women’s health, web-based survey

## Abstract

Unhealthy behaviors may contribute to the development and the progression of chronic diseases with negative consequences on patients’ quality of life. The present study aimed to investigate the relationship between unhealthy behaviors (physical inactivity, tobacco consumption, and alcohol consumption) and health-related quality of life, measured with the SF-36 questionnaire, in women with endometriosis. To achieve this, data from a previous cross-sectional web survey among Italian adult women were analyzed. A total of 1045 responses were included in the analysis. Among the SF-36 subscales, the lowest score was recorded in the energy/fatigue domain: mean = 35.536 (Standard Deviation = 18.452). Smoking and physically inactive women had lower scores than non-smoking and physically active women, respectively, in each SF-36 domain. Women who drank more than one unit of alcohol a day, on average, reported lower scores than women with an alcohol intake <1 unit a day, for the following SF-36 domains: role limitations due to physical health, role limitations due to emotional problems, and emotional well-being. The multivariable analysis evidenced that employment, physical inactivity, and tobacco consumption were significant predictors for each SF-36 domain (*p* < 0.05). Physical inactivity and tobacco consumption had negative effects on the SF-36 subscales. Our results showed the need to monitor unhealthy behaviors to improve the overall well-being of women with endometriosis. Tailored strategies addressing smoking cessation, promoting physical activity, and moderating alcohol intake may aid in enhancing health-related quality of life in this vulnerable population.

## 1. Introduction

Endometriosis is a chronic and often debilitating condition that affects an estimated 10–15% of women of reproductive age worldwide [1]. It is characterized by the growth of endometrial-like tissue outside the uterus, which can cause severe pain, infertility, and a range of other symptoms [2]. Affected individuals also have an increased risk of non-reproductive sequelae, such as high blood pressure, cardiovascular disease, autoimmune conditions, gastrointestinal and urologic symptoms, multi focal pain, migraines, and ovarian and breast cancer. These symptoms are primarily linked to life-altering pelvic pain, painful menstruation and sexual relations, infertility, exhaustion, and depression [3]. According to Martire et al., the presence of different US signs of endometriosis (DIE, endometrioma, and adenomyosis) is significantly lower in the 12–16 years subgroup than in the 21–25 years subgroup. This data may confirm that endometriosis worsens with more menstrual cycles [4].

This evidence is also supported by the experience of Millischer et al. In their study, the prevalence of endometriosis increases with age, with a significant threshold at the age of 18 [5].

Some authors have investigated the possible role of environmental or occupational risk factors. According to epidemiological data, organochlorine exposure plays a role as a risk factor for endometriosis development, as well as night shift work [6].

Risk factors that increase the risk of endometriosis include the consumption of products rich in trans-unsaturated fatty acids, the consumption of fats generally, and the consumption of red meat and alcohol [7]. Early age at menarche and short menstrual cycle length are associated with an increased risk, while parity and current oral contraceptive use are associated with a decreased risk [8]. The study of Shah et al. confirms the robust inverse association between body size and endometriosis [9]. According to Aarestrup et al., childhood BMI seems to be inversely associated with endometriosis. In contrast, childhood height seems to be positively associated with endometriosis [10].

Endometriosis can significantly affect a woman’s social and physical well-being [11,12], which adds to the disease’s cost in terms of its effects on quality of life (QoL) and its influence on the economic aspects. Moreover, endometriosis raises the risk of missing work or school [13]. The illness has a substantial impact on women’s social activities, sexual relationships, and mental and emotional health [14,15,16]. Scientific research has shown that endometriosis can lower physical QoL to a level comparable to the physical QoL perceived by cancer patients [14,17].

### 1.1. Endometriosis and Unhealthy Behaviors

While the exact cause of endometriosis is unknown, it is thought to be due to a combination of genetic, hormonal, environmental, and lifestyle factors [18,19]. In particular, emerging evidence suggests a potential relationship between certain unhealthy behaviors and the development or exacerbation of this condition. Several studies have explored the impact of diet on endometriosis: a meta-analysis by Missmer et al. [20] found that diets with high percentages of trans fats and red meat were associated with an increased risk of endometriosis. Conversely, a diet rich in omega-3 fatty acids, as demonstrated in the work of Trabert et al. [21], showed a potential protective effect against endometriosis development.

Regular physical activity seems to have a protective effect on diseases that involve inflammatory processes and oxidative stress, such as endometriosis, as it increases systemic levels of anti-inflammatory cytokines. In a case-control study, increased physical activity levels were associated with a lower incidence of endometriosis. These findings emphasize the potential benefits of regular exercise in mitigating the risk of developing endometriosis [22].

A comprehensive review by Kvaskoff et al. [23] explored the association between smoking and endometriosis. The review revealed a potential link between cigarette smoking and an increased risk of endometriosis, underlying the importance of tobacco cessation in individuals at risk of developing endometriosis. However, in women with endometriosis, there were no differences in smoking among the various groups based on disease extension and grade (SUP, OMA, and DIE) [24].

Long-term alcohol consumption also appears to be associated with an increased risk of chronic inflammatory diseases by regulating the production of pro-inflammatory cytokines. Parazzini et al. found an alcohol dose-dependent increase for the risk of endometriosis, as heavy drinkers showed a higher risk [25]. According to the study of De Angelis et al., passive smoking during childhood and alcohol consumption might increase the risk of developing endometriosis. With regard to alcohol consumption, there are significant differences between current consumers and former consumers, fertility status, and differential diagnosis of deep endometriotic nodules vs. peritoneal disease. The risk of occurrence of deep endometriosis nodules is higher than the risk of peritoneal nodules; furthermore, the increased risk was found in patients who were current drinkers of alcohol, and not in infertile patients. The same study suggests that current smokers’ natural fertility decreases [26].

### 1.2. Unhealthy Behaviors and Health-Related QoL (HR-QoL)

Concerning the relationships between unhealthy behaviors and health-related QoL (HR-QoL), the negative impact of smoking on health-related quality of life is well documented in the general population and in the unhealthy population. A recent study investigated the effects of smoking cessation on quality of life, showing the positive effect on physical aspects of HR-QoL [27]. Taylor et al. recently found that smoking cessation had a positive impact on reducing anxiety and depression symptoms [28].

The literature well supports the important benefit of physical activity, both in the prevention of some diseases (excess weight, cardiovascular disease, type 2 diabetes, chronic pain, and some types of cancer) in different populations [29], and in the improvement of HR-QoL [30].

Some authors, using different instruments, have also investigated the relationship between alcohol and HR-QoL, and recently Redwood and colleagues reported a significant association only in users with severe risk of alcohol disorder [31].

### 1.3. Short Form Health Survey 36 (SF-36) and Endometriosis

Among the tools that evaluate health-related quality of life, the SF-36 questionnaire is the most frequently used and it is one of the most widely generic measures of health, commonly used in studies of endometriosis [32,33].

As reported by Bestel and colleagues, women with endometriosis report lower mean scores than healthy women in all quality-of-life domains of the SF-36 questionnaire [34], and endometriosis was found to be associated with worse QoL over time [35].

Investigating in a clinical setting the relationship between QoL measured using the SF-36 tool and unhealthy behaviors, a recent study evidenced that physical inactivity was significantly associated with all the SF-36 subscales, and the habit of smoking was significantly related to mental QoL [36].

### 1.4. Unhealthy Behaviors, Health-Related QoL, and Endometriosis

Based on the premises of the literature above, it is hypothesized that, among the well-known etiopathological factors bearing on the development of endometriosis, unhealthy behaviors may lead to the development of endometriosis. In addition, the consequent health-related QoL could impact the general QoL, creating a vicious cycle. In this cycle, several predisposing factors act as precursors, while pathophysiological conditions, together with unhealthy behaviors, act as etiological factors. The result is that women can show low health-related QoL. The evaluation of all these aspects may help the clinician to diagnose endometriosis, and also to treat women from the point of view of unhealthy behaviors. The literature shows that in the field of unhealthy behavior prevention, some specific strategies, called “harm reduction” (HR) strategies, are often useful for the management of the risk associated with the perpetration of those behaviors [37]. In this perspective, policies and community-based measures based on HR could reduce the risk of the genesis of disease, or reduce the impact of diseases on QoL, giving people the possibility of continuing with potentially hazardous behaviors. Hence, the individuation of unhealthy behaviors associated with endometriosis could permit the adoption of HR strategies, reducing the negative impact of these variables on health-related QoL.

To our best knowledge, there have been no studies in Italy on the relationships between unhealthy behaviors and the SF-36 subdomains in women with endometriosis. The present study aimed to investigate the relationship between the SF-36 subdomains and clinical sociodemographic factors, and unhealthy behaviors (physical inactivity, tobacco smoking, and alcohol consumption) in women with endometriosis.

## 2. Materials and Methods

The present study represents a secondary analysis of a data set from the project “Quality of care and perception of quality of life in women with endometriosis in the covid era”, the results of which have already been partially reported [17,38].

This secondary analysis was focused on the relationship between unhealthy behaviors (physical inactivity, tobacco smoking, and alcohol consumption) and eight domains or subscales of QoL (physical functioning, role limitations due to physical health, energy/fatigue, social functioning, general health, role limitations due to emotional problems, emotional well-being) in women with endometriosis.

### 2.1. Study Design and Population

It was a cross-sectional study. Italian-speaking women aged 18 years or more, with a self-reported diagnosis of endometriosis, were eligible for the study. Participants provided informed consent before being accepted to the online survey, filling out an anonymous questionnaire via Google Forms. The survey participation was optional from July to September 2021, and participants were free to leave at any moment.

### 2.2. Procedures and Measures

The adopted questionnaire consisted of three parts.

The first section consisted of items to collect sociodemographic data and information about healthy habits (nationality, age, residence, marital status, living alone, employment, educational level, tobacco smoking, physical activity, and alcohol consumption).

The second section contained clinical data, including age at the moment of the endometriosis diagnosis, pregnancy, height and weight, time from symptoms to diagnosis, treatments, complications related to endometriosis, and comorbidities.

The third part of the questionnaire was dedicated to evaluating the perceived quality of care, and the quality of life (QoL), as follows.

Modified items from a validated questionnaire for quality-of-care evaluation of the diabetic population [39] were used to investigate the quality of the healthcare unit and the healthcare workers who had taken care of women suffering from endometriosis during the last 12 months. Respondents rated the healthcare unit that had provided primary care for them during the past 12 months on the following items: opening hours of the structure; accessibility of the rooms; cleanliness and agreeability; courtesy and helpfulness; understandable explanations; being listened to; waiting times from booking to the visit; waiting time from arrival to the hospital/clinic; waiting time from arrival to visit; overall rating of the service offered in the last 12 months; judgment on the level of coordination between all services and professionals who deal with the disease. Each item had four options, two of which were positive, like “excellent/good” or “adequate/excellent”, and two of which were less favorable, like “not adequate, just adequate” or “not sufficient, sufficient” (see Supplementary Material).

To evaluate health-related quality of life (HR-QoL), the Italian version of the SF-36 questionnaire was used [40]. The SF-36 is a self-administered questionnaire used to assess the impact of a disease on perceived physical and mental health. It consists of 36 items that can be divided into 8 different scales, which conceptually refer to 8 health domains: physical functioning—PF; role limitations due to physical health—RLE; energy/fatigue—EF (vitality subscale); social functioning—SF; pain—P; general health—GH; role limitations due to emotional problems—RLP; and emotional well-being—EW [41]. All questions (except for the last one) assessed the period related to four weeks before filling out the questionnaire. The last item assessed the change in health status from the previous year. A high score corresponds to better health status.

Based on literature evidence, reliability data for each SF-36 subscale related to women with endometriosis are: 0.88 for physical functioning, 0.83 for role physical, 0.79 for bodily pain, 0.73 for general health, 0.82 for vitality, 0.80 for social functioning, 0.81 for role emotional, 0.87 for mental health, 0.89 for the physical health component, and 0.92 for the mental health component [33].

The survey was carried out in 2021 when health services in Italy were reorganized after the lockdown period due to the COVID-19 pandemic [42,43,44]. Specific items about the perceived impact of the COVID-19 pandemic on healthcare quality were also added.

A snowball sampling technique was used, and the sample size was estimated as 1032 units based on 3% precision, considering a 95% confidence interval and a response level for a single parameter of 50%. During the recruitment period, 1065 participants were registered.

### 2.3. Variable Selection

#### 2.3.1. Sociodemographic Variables

The following variables were selected for the present analysis: age, education, marital status, employment, education, and living alone. Age was classified as 18–34 years, 25–44 years, and 45 years or older. Education was synthesized into two categories: “low” (if the educational level reported was an elementary school certificate, middle school certificate, or Diploma) and “high” (if the educational level reported was secondary school or Postgraduate). Marital status was synthesized into two categories: single or widow, and married or cohabiting. Employment was based on the answer to “Do you currently have a job?”, and it was treated as a dichotomous variable: “yes” (if the answer was “Yes, indefinitely” or “Yes, for a fixed term”) and “no” (if the answers were “No, I’m unemployed”, “No, I’m a student”, “No, I’m a housewife”, or “No, I’m retired”). The variable “living alone” was dichotomous (yes/no) based on the item “Do you live alone?”.

#### 2.3.2. Clinical Variables

Regarding clinical information, we considered the duration of the disease and the presence of other diseases. The disease duration (years) was considered as a continuous variable calculated from the difference between the age reported when endometriosis was diagnosed, and the age reported during the survey. It was classified as 0–3 years, 4–9 years, and ≥10 years. The presence of other diseases was encoded as “comorbidities (yes/no)” based on the answer to the item “Do you suffer from other pathologies besides endometriosis?”.

#### 2.3.3. Lifestyles Variables

Physical activity, tobacco smoking habits, and alcohol consumption were selected to investigate unhealthy behaviors.

We used the dichotomous variable “physical inactivity: yes/no”, based on the answers to the item “Do you do physical activity?”. It was categorized as “yes” (if the reported answers were “I am not currently physically active” or “I have never exercised in my life physical activity”) and as “no” (if the reported answers were “Yes, moderate physical activity” or “Yes, intense physical activity”). The alcohol consumption consisted of two questions. The first question was “Do you ever drink a unit of alcohol (1 glass of beer or 1 glass of wine or spirits)?”, and we synthesized it in the variable “alcohol intake (Yes/No)”. It was categorized as “Yes” (if the reported answers were “Yes, only during meals”, “Yes, mainly during meals”, “Yes, mainly between meals”, or “Yes, only between meals”), and as “No” (if the reported answer was “I don’t drink alcohol”). The second question was related to the number of alcohol units consumed: “In a day, how many units of alcohol (1 glass of beer or 1 glass of wine or spirits) do you drink on average? From the two previous questions, we elaborated the dichotomous variable “Alcohol intake >1 drink a day on average”. It was categorized as “Yes” (if the reported alcohol intake was higher than 1 drink per day on average) and as “No” (if the reported alcohol intake was lower than or equal to 1 drink per day on average). This variable was selected to consider “non-moderate alcohol consumption”; in fact, women who consume 1 unit of alcohol per day (which is equivalent to 30 alcohol units consumed on average in the last 30 days), have a moderate alcohol consumption [45]. The variable “tobacco smoking” was derived from the item “Smoking habit: currently”, and it was categorized as “Yes” (if the reported answer was “I smoke cigarettes (tobacco)) and “No” (if the reported answers were “I don’t smoke, but I have smoked in the past” or “I have never smoked”).

#### 2.3.4. Quality of Life Variables

Quality of life evaluation was based on eight continuous variables, which were as follows: physical functioning—PF; role limitations due to physical health—RLE; energy/fatigue—EF (vitality subscale); social functioning—SF; pain—P; general health—GH; role limitations due to emotional problems—RLP; and emotional well-being—EW. The variables were calculated from the scoring of the SF-36 questionnaire: PF (10 items), RLE (3 items), EF (4 items), SF (2 items), P (2 items), GH (5 items), RLP (4 items), and EW (5 items). The eight QoL subdomains are rated from 0 to 100 so that higher values correspond to better conditions.

### 2.4. Data Analysis

For the present analysis, 1045 records were analyzed. Pregnant women (*n* = 20) were removed from the dataset, because of the known relation between pregnancy and quality of life [46]. In particular, during the early 2nd trimester, quality of life is optimal, but as the late 3rd trimester progresses, it decreases as a result of multiple physical and psychological changes [47].

All variables were analyzed and reported as frequencies or mean with standard deviation (SD). Data were tested for normality with the Kolmogorov–Smirnov test and variance homogeneity with the Levene test.

A bivariate analysis was performed to investigate the association between each SF-36 QoL subscale and sociodemographic, clinical, and lifestyle variables.

The *t*-test for independent samples or the U-Mann–Whitney test was used for dichotomous factors calculating effect size for the U statistic with the Kerby formula [48].

The Kruskal–Wallis test was used when the independent factors had more than two categories (age and disease duration).

Then, eight separate multivariable robust regression models were performed corresponding to the eight SF-36 dimensions: physical functioning (PF), role limitations due to physical health (RLP), role limitations due to emotional problems (RLE), energy/fatigue (EF), emotional well-being (EW), social functioning (SF), pain (P), and general health (GH).

Each multivariable regression model was performed according to the study objective. The demographic variables—age (years), marital status (single or widows/married or cohabiting), living alone (yes/no), employment (yes/no), and educational level (low/high)—were entered to control for confounding. The clinical variables of disease duration (years) and comorbidities (yes/no) were entered because other researchers have shown that these are associated with lower QoL [49,50,51]. The lifestyle variables—physical inactivity lifestyle (yes/no), alcohol intake >1 drink a day on average (yes/no), and tobacco smoking (yes/no)—were entered because they served as new elements to be explored in this article.

The significance level was set at 0.05, and all analyses were performed using STATA MP 14 software.

### 2.5. Ethical Considerations

The present study was authorized by the Internal Review Board (IRB) of the University of L’Aquila (Protocol number 26/2021) and informed consent was obtained from all subjects involved in the study.

## 3. Results

Data from a total of 1045 women with endometriosis were examined, with 98% of the women being of Italian nationality. Table 1 summarizes the main characteristics of the investigated sample. In total, 47% of women were aged between 18 and 24 years, and most of the participants (612; 58.6%) were married or cohabiting. Most participants had a low level of education (589; 56.4%), were working (677; 64.8%), and reported not exercising regularly (615; 58.9%). A third of the participants (344; 33.0%) reported not consuming alcohol and about 21% (219) were smokers. More than half of the participants (534; 51.1%) reported a duration of the disease less than 3 years, while 22% of the participants had been affected by endometriosis for 10 years or more. A total of 54.9% (574) of the participants reported having at least one comorbidity.

Considering the eight domains of the SF-36 questionnaire, the participants reported the highest values in the domain of physical functioning, 66.507 (SD = 27.241), and the lowest value in the domain of energy/fatigue, 35.536 (SD = 18.452).

Table 2 reports the mean scores of the SF-36 domains calculated by each considered behavior. Concerning smoking habits, the results indicate lower mean scores for each SF-36 subdomain; in particular, the lowest result was for role limitations due to emotional problems (mean = 31.849, SD = 37.234). The mean score tested by bivariate analysis indicated significant differences between smokers and non-smokers for all domains. Physically inactive women reported scores lower than physically active women and all differences were found to be statistically significant. There were no significant differences between groups by alcohol intake. Women with high alcohol intake (intake >1 unit a day on average), had lower scores for the following domains: role limitations due to physical health—RLP (mean = 35.088, SD = 40.970); role limitations due to emotional problems—RLE (mean = 37.500, SD = 42.215); and emotional well-being—EW (mean = 44.421, SD = 21.984).

Table 3 reports a bivariate analysis performed between all SF-36 domains and the sociodemographic and clinical data. The analysis showed that the perceived quality of life increased on average in older women for the subscales of physical functioning (*p* < 0.001) and pain (*p* = 0.030), while the emotional well-being decreased from the younger to the older women investigated (*p* = 0.004). Marital status had a significant effect on role limitations due to physical health (mean score in married or cohabiting women = 41.9 versus mean score in single women or widows = 36.6) and social functioning (mean score in married or cohabiting women = 45.8 versus mean score in single women or widows = 42.7) subscales. The SF-36 subscales of physical functioning, pain, general health, and role limitations due to physical health were influenced by a high educational level and a better quality of life. The quality of life was not impacted by living alone, despite the significant impact of employment on all SF-36 domains. In all the domains investigated, women who had comorbidities had a worse quality of life and lower scores than women without comorbidities, according to the clinical data. All subscales except for physical functioning and general health were significantly impacted by disease duration.

As reported in Table 4, alcohol consumption, civil status, and living alone were not significantly related to the SF-36 subscales, even though the coefficient beta between the pain domain and alcohol consumption was 9.07 (*p* = 0.054). The multivariable analyses evidenced that among the sociodemographic variables, age was significantly associated with physical functioning (beta= −0.78, *p* < 0.001), the vitality subscale (energy-fatigue) (beta = 0.19, *p* = 0.039), pain (beta = −0.45, *p* = 0.002), and role limitations due to emotional problems (beta = −0.50, *p* = 0.017). Occupational status was a significant predictor for all SF-36 domains, indicating that employed women perceived better QoL. Educational level was positively associated with physical functioning (beta = 9.45, *p* < 0.001), pain (beta = 5.34, *p* = 0.004), and role limitations due to emotional problems (beta = 9.36, *p* < 0.001).

Physical inactivity and smoking habits were significantly related to all SF-36 domains, indicating their negative impact on all domains of QoL in the target population.

For the investigated clinical characteristics, the regression analysis showed that disease duration was statistically negatively related to social functioning (beta = 0.46, *p* = 0.002), pain (beta = 0.71, *p* < 0.001), role limitations due to physical health (beta = 0.79, *p* = 0.002), and emotional well-being (beta = 0.22, *p* = 0.048). As expected, the presence of other diseases (comorbidities) was a predictor of worse QoL and it was related to all the QoL domains.

## 4. Discussion

Our study sheds light on the intricate relationship between unhealthy behaviors, health-related QoL, and their impact on endometriosis. Based on the SF-36 subdomains, we found important data about the physical and mental QoL of women affected by endometriosis. The sample investigated reported scores of SF-36 subdomains ranging from the minimum mean score of 35.536 (SD = 18.45), collected for the energy/fatigue domain, to the maximum mean score of 66.507 (SD = 27.241) for the physical functioning domain. These data were in line with Bestel and colleagues, even if the other subscale scores of our study were lower [34].

The physical functioning and role limitations due to emotional problems subdomains were positively related to employment and educational level, and were negatively related to age, physical inactivity, and the presence of comorbidities. Role limitations due to physical health (mean score 40.255), social functioning (mean score 44.282), general health (mean score 37.091), and emotional well-being (mean score 47.567) were positively related to employment and were negatively related to physical inactivity and the presence of comorbidities. Our study found positive associations between employment status and higher education level with several subdomains of the SF-36. These results may be related to the fact that high instruction and resource availability help patients to access the best health care, affecting the quality of their lives. It has been reported that women with endometriosis are more satisfied with the quality of the care they receive from specialized centers [38]. A high income also allows easier access to pharmacological, physiotherapeutic, and psychological treatments not provided by the National Health Service (NHS). All these aspects could lead, in women with lower sociocultural levels, to increased diagnostic delays or otherwise avoidable complications, affecting their QoL [52]. Such inequalities in the Italian health system are unfortunately well known and represent a major challenge for the NHS, which should try to reduce them, for the wellness of Italian citizens and the health system itself [53,54].

In addition, role limitations due to emotional problems, social functioning, and emotional well-being were positively related to disease duration. In our study, the comorbidity factor was found to be a significant predictor of poor QoL, while disease duration was a positive predictor of QoL for some investigated SF-36 subdomains. As reported in previous studies, endometriosis is associated with gynecological (adenomyosis, uterine fibroids, and polycystic ovarian syndrome—PCOS) and systemic comorbidities, including immune (asthma, rheumatoid arthritis, psoriasis, and multiple sclerosis), inflammatory (bowel inflammatory disease and Crohn’s disease), and psychiatric disorders (depression and anxiety). All these aspects impair women’s QoL, influencing everyday life and work activities [55]. Conversely, the impact of disease duration on quality of life would seem inconsistent in endometriosis [56].

The energy/fatigue subdomain was positively related to age and employment, and it was negatively related to physical inactivity and the presence of comorbidities. These associations give clinicians a possible explanation of why pain and fatigue can tend to become chronic in these patients, following the biopsychosocial model [57].

Another interesting result is the positive association between the SF-36 pain subdomain and the following variables: educational level and duration of disease. On the contrary, the pain subdomain was negatively associated with age, physical inactivity, smoking, comorbidities, age, and employment. In the literature, it has been evidenced that pain severity in women suffering from endometriosis was positively associated with subjective psychosocial impairment [58]. Psychological interventions for the management of pain have been instead documented as efficient [59,60]. The meaning of this literature evidence is that pain, as well as fatigue, has important psychological components that should always be assessed and treated when endometriosis is diagnosed. Psychological well-being is a multifactorial aspect that can be influenced by several variables. Among these variables, physical activity and healthy lifestyles can fight the insurgence of comorbidities, chronic pain, and fatigue, giving women the possibility of living with endometriosis to the best of their abilities. In Dybowski et al.’s study, the duration of chronic pelvic pain syndrome (CPPS) symptoms was not associated with any of the outcomes (pain intensity, urinary symptoms, and impediments to quality of life). According to the authors, a plausible explanation for this may refer to the chronicity of the CPPS-specific symptoms. They found significant improvements in pain intensity, impediments to quality of life, and health anxiety over the 12 months, probably thanks to the multidisciplinary approach to these patients (psychologists or psychotherapists and physical therapists) [61]. During the assessment of women with suspected endometriosis, it is important to keep in mind that younger age, physical inactivity, the presence of comorbidities, and smoking can worsen QoL, causing women to not have the resources they need for managing the endometriosis-related pain.

Another finding of the study was that the scores of the SF-36 subscales were significantly higher in the group with a high alcohol intake, even though there were no significant differences in the bivariate analyses. The multivariable analysis confirmed that alcohol intake was not a significant predictor of QoL in women with endometriosis. This result is partly in contrast with the literature evidence. In fact, a recent meta-analytic study found a positive association between alcohol intake and endometriosis, suggesting that alcohol could be considered on one hand a coping strategy for the management of several difficulties related to endometriosis, and, on the other hand, it could directly increase the risk of developing endometriosis through inflammatory mediators and oxidative stress [62]. Hence, based on these contradictory findings, it is only possible to suppose that alcohol intake is an indicator of women’s general QoL.

The bivariate analysis of each SF-36 QoL subscale also showed that the mean scores were predominantly lower in women who reported smoking habits and physical inactivity. The multivariable analysis confirmed that smoking habits and physical inactivity were significantly related to all SF-36 subdomains. The analysis evidenced that adjusting for all the analyzed factors, a smoking habit negatively impacted all the QoL domains, in particular the role limitations due to physical health (RLE) subscale, with beta = −10.59 and *p* = 0.002.

Therefore, physical inactivity was a predictor of poor QoL, and it was particularly important in physical functioning, with beta = −7.52 and *p* < 0.001.

These data support the significant impact that some unhealthy behaviors, among them alcohol abuse and smoking habits, may have an association with the disease. In fact, the first important finding shows that 38 women with a self-referred diagnosis of endometriosis reported drinking more than an alcohol unit a day on average. The proportion of women with strong alcohol consumption was 5% among women who reported drinking (38/701) and 3.6% among all the participants (38/1045). This proportion was the same as the data from the National Observatory for Alcohol in Italy on habitual excess consumption in women aged from 18 to 49 years (3.6%) [63].

The proportion of women who reported smoking tobacco was in line with the literature (21%). In chronic disease, the smoking proportion was documented as ranging from 15% to 26% [64]. More than half of the participants (59%) reported physical inactivity, and as reported in other studies, it seems that women with endometriosis engage in physical activity less than healthy women [65].

The study presents some limitations, mainly related to the fact that it was based on patients’ self-reported information. We do not know the psychological profile of the participants or the severity of endometriosis that was found to be associated with quality of life [66].

## 5. Conclusions

The findings underscore the nuanced impact of specific behaviors, such as smoking, physical inactivity, and alcohol consumption, on the various domains of the SF-36 questionnaire. Notably, our results reveal that smoking is consistently associated with lower scores across all SF-36 domains, emphasizing the pervasive negative effects of tobacco consumption. Furthermore, physical inactivity emerged as a significant predictor, providing valuable insights into the multifaceted nature of these lifestyle factors.

The multivariable analyses highlight the importance of considering employment status, physical activity levels, and tobacco consumption when assessing health-related quality of life in women with endometriosis. In addition, pain and fatigue can also be managed in women suffering from endometriosis by considering their lifestyle habits, including the limitation of smoking and the promotion of physical activity. These factors emerged as robust predictors across all SF-36 domains, emphasizing their relevance in understanding and addressing the complexities of health outcomes in this population.

In light of these findings, our study underscores the need for comprehensive interventions that target specific unhealthy behaviors to improve the overall well-being of women with endometriosis. Tailored strategies addressing smoking cessation, promoting physical activity, and moderating alcohol intake may contribute to enhancing health-related quality of life in this vulnerable population. Further research and targeted interventions are warranted to refine our understanding of these associations and inform more effective approaches to support the holistic well-being of individuals living with endometriosis.

## Figures and Tables

**Table 1:** Characteristics of the study population.

Variables	*n* (%) or Mean (SD)
Age groups, *n* (%)	
18–34 years	491 (47.0%)
35–44 years	435 (41.6%)
≥45 years	119 (11.4%)
Marital status, *n* (%)	
Single or widows	433 (41.4%)
Married or cohabiting	612 (58.6%)
Education level, *n* (%)	
Low	589 (56.4%)
High	456 (43.6%)
Employment, *n* (%)	
No	368 (35.2%)
Yes	677 (64.8%)
Living alone, *n* (%)	
No	931 (89.1%)
Yes	114 (10.9%)
Physical inactivity, *n* (%)	
No	430 (41.1%)
Yes	615 (58.9%)
Alcohol intake, *n* (%)	
No	344 (33.0%)
Yes (only during meals; Yes, mainly during meals; Yes, mainly between meals; or Yes, only between meals)	701 (67.0%)
If yes: In a day, how many units of alcohol (1 glass of beer or 1 glass of wine or spirits) do you drink on average?	
<1	436 (62.20%.)
1	227 (32.38%)
2	32 (4.56%%)
3	3 (0.4%)
4	3 (0.4%)
Tobacco smoking, *n* (%)	
No	826 (79.0%)
Yes	219 (21.0%)
Disease duration, *n* (%)	
0–3 years	534 (51.1%)
4–9 years	276 (26.4%)
≥ 10 years	235 (22.5%)
Comorbidities, *n* (%)	
No	471 (45.1%)
Yes	574 (54.9%)
SF-36: HR-QoL domains, mean (SD)	
Physical functioning (PF)	66.507 (27.241)
Role limitations due to physical health (RLE)	40.255 (41.136)
Energy/fatigue (EF)	35.536 (18.452)
Social functioning (SF)	44.282 (25.531)
Pain (P)	46.244 (27.763)
General health (GH)	37.091 (20.732)
Role limitations due to emotional problems	39.258 (38.919)
Emotional well-being (EW)	47.567 (19.185)

**Table 2:** Mean score of SF-36 subscales by behaviors.

SF-36: HR-QoL Domains	Unhealthy Behaviors					
	Tobacco Smoking		Physical Inactivity		Alcohol Intake >1 Drink a Day	
	No *n* = 826	Yes *n* = 219	No *n* = 430	Yes *n* = 615	No *n* = 1007	Yes *n* = 38
Physical functioning (PF)	68.269 (26.793)	59.863 (27.949)	72.907 (24.887)	62.033 (27.934)	66.410 (27.319)	69.079 (25.305)
	ES = 0.179		ES = 0.236		ES = 0.043	
	*p* < 0.001		*p* < 0.001		*p* = 0.654	
Role limitations due to physical health (RLP)	42.413 (41.548)	32.116 (38.559)	44.806 (41.814)	37.073 (40.385)	40.450 (41.149)	35.088 (40.970)
	ES = 0.136		ES = 0.138		ES = 0.108	
	*p* = 0.001		*p* = 0.003		*p* = 0.381	
Energy/fatigue (E/F)	36.235 (18.290)	32.900 (18.859)	39.686 (18.406)	32.634 (17.936)	35.457 (18.409)	37.632 (19.685)
	ES = 0.110		ES = 0.215		ES = 0.055	
	*p* = 0.012		*p* < 0.001		*p* = 0.564	
Social functioning (SF)	45.369 (25.311)	40.183 (25.997)	47.558 (25.387)	41.992 (25.402)	44.079 (25.238)	49.671 (32.295)
	ES = 0.121		ES = 0.120		ES = 0.088	
	*p* = 0.005		*p* < 0.001		*p* = 0.351	
Pain (P)	47.582 (27.419)	41.199 (28.525)	50.116 (28.010)	43.537 (27.286)	45.946 (27.629)	54.145 (30.438)
	ES = 0.130		ES = 0.138		ES = 0.161	
	*p* = 0.002		*p* < 0.001		*p* = 0.091	
General health (GH)	38.015 (20.697)	33.607 (20.538)	40.698 (21.518)	34.569 (19.794)	36.946 (20.690)	40.921 (21.743)
	ES = 0.128		ES = 0.171		ES = 0.102	
	*p* = 0.003		*p* < 0.001		*p* = 0.285	
Role limitations due to emotional problems (RLE)	41.223 (39.140)	31.849 (37.234)	44.826 (39.677)	35.366 (37.929)	39.325 (38.810)	37.500 (42.215)
	ES = 0.134		ES = 0.104		ES = 0.079	
	*p* = 0.001		*p* < 0.001		*p* = 0.585	
Emotional well-being (EW)	48.450 (19.054)	44.237 (19.355)	50.502 (18.752)	45.515 (19.233)	47.686 (19.074)	44.421 (21.984)
	ES = 0.127		ES = 0.158		ES = 0.108	
	*p* = 0.004		*p* < 0.001		*p* = 0.257	

ES = effect size calculated by Kerby Formula; *p* from Mann–Whitney test.

**Table 3:** Univariate analysis for each SF-36 domain.

	PF	RLE	EF	SF	P	GH	RLP	EW
Variables	Mean (SD)	Mean (SD)	Mean (SD)	Mean (SD)	Mean (SD)	Mean (SD)	Mean (SD)	Mean (SD)
Demographic factors								
Age groups, *n* (%) **								
18–34 years	71.0 (25.6)	37.0 (40.7)	34.4 (17.9)	44.0 (25.8)	48.6 (27.8)	37.6 (21.3)	41.6 (40.0)	45.6 (18.9)
35–44 years	63.8 (27.4)	42.7 (41.2)	36.3 (18.2)	45.0 (24.9)	45.1 (27.4)	36.1 (20.0)	37.0 (37.7)	49.4 (18.8)
≥45 years	57.7 (30.1)	44.5 (42.4)	37.7 (21.4)	44.4 (27.1)	42.6 (28.3)	38.6 (21.2)	37.8 (38.6)	49.0 (20.9)
	ES = 0.03	ES = 0.01	ES = 0.00	ES = 0.00	ES = 0.01	ES = 0.00	ES = 0.00	ES = 0.01
	*p* < 0.001	*p* = 0.033	*p* = 0.144	*p* = 0.808	*p* = 0.030	*p* = 0.324	*p* = 0.267	*p* = 0.004
Marital status, *n* (%)								
Single or widow	67.6 (27.1)	36.6 (39.9)	34.8 (18.8)	42.7 (25.8)	46.9 (28.3)	36.5 (20.4)	37.9 (38.4)	46.3 (19.6)
Married or cohabiting	5.7 (27.4)	42.8 (41.9)	36.1 (18.2)	45.8 (25.3)	46.2 (27.3)	37.5 (21.0)	40.2 (39.3)	48.4 (18.9)
	ES = 0.04	ES = 0.08	ES = 0.03	ES = 0.08	ES = 0.02	ES = 0.01	ES = 0.03	ES = 0.11
	*p* = 0.250	*p* = 0.023	*p* = 0.344	*p* = 0.023	*p* = 0.613	*p* = 0.773	*p* = 0.393	*p* = 0.081 *
Education level, *n* (%)								
Low (secondary school or lower)	60.7 (27.7)	39.6 (41.6)	34.9 (18.4)	44.5 (25.4)	43.4 (27.3)	35.2 (20.4)	34.2 (37.7)	47.0 (19.3)
High (degree or above)	73.9 (24.8)	41.0 (40.6)	36.4 (18.5)	44.5 (25.7)	50.4 (27.9)	39.6 (21.0)	45.8 (39.6)	48.3 (19.0)
	ES= 0.28	ES = 0.03	ES = 0.04	ES = 0.00	ES = 0.15	ES = 0.13	ES = 0.17,	ES = −0.07
	*p* < 0.001	*p* = 0.383	*p* = 0.246	*p* = 0.986	*p* < 0.001	*p* < 0.01	*p* < 0.001	*p* = 0.279 *
Employment, *n* (%)								
No	61.1 (28.7)	33.0 (41.6)	32.0 (17.7)	40.4 (25.0)	42.7 (27.9)	33.0 (20.1)	31.8 (38.9)	43.8 (18.9)
Yes	69.5 (25.9)	44.2 (40.4)	37.4 (18.6)	46.7 (25.5)	48.5 (27.4)	39.3 (20.8)	43.3 (38.4)	49.6 (19.0)
	ES = 0.17	ES = 0.17	ES = 0.16	ES = 0.14	ES = 0.12	ES = 0.18	ES = 0.19	ES = 0.17
	*p* < 0.01	*p* < 0.01	*p* < 0.01	*p* < 0.01	*p* < 0.01	*p* < 0.01	*p* < 0.01	*p* < 0.01
Living alone, *n* (%)								
No	66.3 (27.4)	40.4 (41.6)	35.4 (18.5)	44.7 (25.3)	46.6 (27.6)	37.1 (20.9)	39.2 (39.0)	47.3 (19.1)
Yes	68.6 (25.9)	39.1 (37.7)	36.4 (18.2)	42.9 (27.2)	44.9 (28.8)	37.2 (19.5)	39.4 (38.2)	49.7 (19.4)
	ES = 0.04	ES = 0.00	ES = 0.05	ES = 0.05	ES = 0.04	ES = 0.01	ES = 0.102	ES = 0.08
	*p* = 0.499	*p* = 0.957	*p* = 0.387	*p* = 0.345	*p* = 0.504	*p* = 0.806	*p* = 0.749	*p* = 0.142
Clinical factors								
Comorbidities, *n* (%)								
No	74.2 (25.2)	43.6 (42.3)	39.3 (17.6)	49.4 (26.2)	53.0 (28.3)	44.2 (20.3)	47.5 (40.0)	49.3 (18.5)
Yes	60.2 (27.3)	37.5 (40.1)	32.5 (18.6)	40.5 (24.3)	41.1 (26.1)	31.2 (19.2)	32.5 (36.7)	46.2 (19.6)
	ES = 0.31,	ES = 0.07	ES = 0.22	ES = 0.19	ES = 0.24	ES = 0.36	ES = 0.2,	ES = 0.09
	*p* < 0.001	*p* = 0.032	*p* < 0.001	*p* < 0.001	*p* < 0.001	*p* < 0.001	*p* < 0.001	*p* = 0.010
Disease duration, *n* (%) **								
0–3 years	67.4 (26.6)	35.5 (40.2)	33.7 (17.7)	41.0 (25.2)	43.6 (27.2)	36.1 (20.4)	35.4 (37.6)	44.9 (18.5)
4–9 years	67.4 (27.5)	47.4 (42.6)	37.7 (18.4)	49.2 (25.0)	50.5 (27.8)	38.4 (21.4)	45.7 (41.3)	50.8 (18.5)
≥10 years	63.4 (28.2)	42.5 (40.5)	37.1 (19.7)	46.7 (25.9)	48.2 (28.2)	37.8 (20.7)	40.4 (38.2)	49.9 (20.6)
	ES = 0.00	ES = 0.01	ES = 0.01	ES = 0.02	ES = 0.01	ES = 0.00	ES = 0.01	ES = 0.02
	*p* = 0.164	*p* < 0.001	*p* = 0.006	*p* < 0.001	*p* = 0.002	*p* = 0.261	*p* = 0.002	*p* < 0.001

ES = effect size from Kerby formula; *p* = *p*-value from Mann–Whitney test; * *p* = *p*-value from *t*-test; ** Kruskall–Wallis test was performed.

**Table 4:** Multivariable regression analysis for each SF-36 domain.

Models		Age	Civil Status	Live Alone	Employment	Educational Level	Physical Inactivity	Tobacco Smoking	Alcol *	Disease Duration	Comorbidities
Model 1: PF R2 = 0.177 F = 22.3 *p* < 0.001	B	−0.78	2.47	0.34	7.41	9.45	−7.52	−6.20	3.89	0.23	−12.44
	95% CI										
	Lower	−1.04	−1.33	−5.42	3.83	6.11	−10.81	−10.15	−4.66	−0.07	−15.66
	Upper	−0.52	6.26	6.11	10.99	12.79	−4.23	−2.23	12.43	0.52	−9.21
	*p*	0.000	0.203	0.907	0.000	0.000	0.000	0.002	0.372	0.130	0.000
Model 2: RLE R2 = 0.047 F = 5.08 *p* < 0.001	B	0.35	2.86	−3.40	10.30	−1.53	−6.47	−10.59	−4.08	0.25	−6.42
	95% CI										
	Lower	−0.10	−3.68	−13.34	3.87	−7.29	−12.13	−17.41	−18.81	−0.25	−11.98
	Upper	0.79	9.40	6.53	16.20	4.22	−0.80	−3.77	10.64	0.76	−0.87
	*p*	0.128	0.391	0.502	0.001	0.601	0.025	0.002	0.586	0.328	0.023
Model 3: EF R2 = 0.092 F = 10.48 *p* < 0.001	B	0.19	0.03	−1.09	3.18	−0.66	−6.01	−3.20	2.11	0.12	−6.98
	95% CI										
	Lower	0.10	−2.65	−5.15	0.65	−3.01	−8.33	−5.99	−3.92	−0.09	−9.25
	Upper	0.38	2.71	2.98	5.70	1.70	−3.69	−0.41	8.14	0.33	−4.70
	*p*	0.039	0.984	0.600	0.014	0.585	0.000	0.025	0.492	0.265	0.000
Model 4: SF R2 = 0.070 F = 7.74 *p* < 0.001	B	−0.09	2.27	−3.92	5.40	−1.84	−4.45	−5.66	5.38	0.46	−8.84
	95% CI										
	Lower	−0.34	−1.48	−9.62	1.87	−5.14	−7.70	−9.57	−3.06	0.17	−12.02
	Upper	–0.17	6.02	1.78	8.94	1.46	−1.20	−1.75	13.82	0.75	−5.65
	*p*	0.498	0.236	0.177	0.003	0.275	0.007	0.005	0.212	0.002	0.000
Model 5: P R2 = 0.098 F = 11.2 *p* < 0.001	B	−0.45	−0.46	−4.95	4.71	5.34	−4.87	−5.79	9.07	0.71	−11.75
	95% CI										
	Lower	−0.73	−4.56	−11.18	0.85	1.74	−8.42	−10.06	−0.15	0.39	−15.23
	Upper	−0.17	3.64	1.27	8.57	8.94	−1.32	−1.52	18.29	1.03	−8.27
	*p*	0.002	0.825	0.119	0.017	0.004	0.007	0.008	0.054	0.000	0.000
Model 6: GH R2 = 0.136 F = 16.3 *p* < 0.001	B	−0.01	0.49	−2.52	4.43	2.41	−4.32	−3.78	3.52	0.19	−12.47
	95% CI										
	Lower	−0.21	−2.41	−6.93	1.69	−0.15	−6.83	−6.81	−3.01	−0.03	−14.93
	Upper	0.18	3.40	1.89	7.17	4.96	−1.80	−0.75	10.06	0.42	−10.00
	*p*	0.894	0.739	0.262	0.002	0.065	0.001	0.014	0.291	0.098	0.000
Model 7: RLP R2 = 0.089 F = 10.1 *p* < 0.001	B	−0.50	3.74	−2.15	9.89	9.36	−6.67	−7.58	−2.38	0.79	−14.66
	95% CI										
	Lower	−0.90	−2.22	−11.21	4.28	4.12	−11.84	−13.80	−15.79	0.27	−19.72
	Upper	−0.09	9.70	6.90	15.51	14.60	−1.51	−1.37	11.03	1.20	−9.60
	*p*	0.017	0.219	0.641	0.001	0.000	0.011	0.017	0.728	0.002	0.000
Model 8: EW R2 = 0.059 F = 6.52 *p* < 0.001	B	0.17	0.90	1.40	4.00	0.03	−4.11	−3.81	−4.05	0.22	−3.26
	95% CI										
	Lower	−0.02	−1.94	−2.92	1.32	−2.47	−6.58	−6.78	−10.46	0.00	−5.67
	Upper	0.36	3.75	5.73	6.69	2.53	−1.64	−0.84	2.36	0.44	−0.84
	*p*	0.085	0.534	0.524	0.004	0.982	0.001	0.012	0.215	0.048	0.008

* Alcohol Intake > 1 Drink a Day; PF = physical functioning; RLE = role limitations due to physical health; E/F = energy/fatigue; SF = social functioning; GH = general health; RLE = role limitations due to emotional problems, and EW = emotional well-being.

## Data Availability

The data analyzed in this study are not available to outside researchers due to privacy issues.

## Supplementary Materials

The following supporting information can be downloaded at: https://www.mdpi.com/article/10.3390/bs14030199/s1, Questionnaire.
