# Supplementary material for: Investigating Unhealthy Behaviors Associated with SF-36 Domains in Women with Endometriosis—Findings from a Web-Based Survey Data Set
Source: Behav Sci (Basel). 2024 Mar 1;14(3):199. doi: 10.3390/bs14030199 (PMC10968121; doi:10.3390/bs14030199)
Supplement: Supplementary file 1 [file behavsci-14-00199-s001.zip › behavsci-2835999-supplementary.pdf]

Qualità dell'assistenza e percezione della qualità della vita nelle donne con endometriosi in era Covid

Il presente questionario è destinato a Donne con età ≥18 anni, con diagnosi di endometriosi

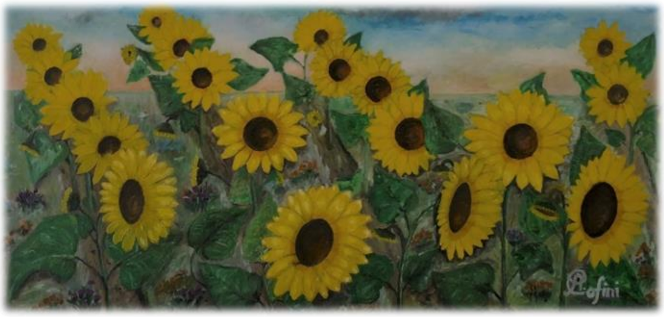

Gentile Signora,

Le proponiamo di partecipare ad un'indagine per valutare l'impatto della Pandemia Covid-19 sulla qualità dell'assistenza ricevuta e sulla qualità della vita delle donne con diagnosi di endometriosi, ad un anno dall'inizio della Pandemia. A tale scopo Le chiediamo la disponibilità di 10 minuti circa, invitandola a rispondere ad un questionario online anonimo. I dati raccolti saranno trattati secondo la normativa vigente per la tutela della privacy, saranno elaborati in forma aggregata non riconducibile ad alcun partecipante e saranno utilizzati per lavori scientifici. La invitiamo pertanto a dare il suo consenso dopo aver letto il foglio informativo sullo studio e la nota informativa sul trattamento dei dati. Potrà interrompere la sua partecipazione in qualsiasi momento, senza bisogno di fornire alcuna spiegazione ottenendo così il NON utilizzo dei dati

Dati anagrafici

D1 – Anni compiuti *

____

D2- Nazionalità *

- o Italiana
- o Altro __________

D3 – Comune di residenza *

____

D4 – Provincia *

Indicare la sigla con lettere maiuscole

____

D5 - Regione di provenienza *

- o Abruzzo
- o Basilicata
- o Calabria
- o Campania
- o Emilia-Romagna
- o Friuli Venezia Giulia
- o Lazio
- o Liguria
- o Lombardia
- o Marche
- o Molise
- o Piemonte
- o Puglia
- o Sardegna
- o Sicilia
- o Toscana
- o Trentino-Alto Adige

- Umbria
- Valle d'Aosta
- Veneto

D6 - Stato civile *
- Coniugata
- Nubile
- Vedova
- Divorziata
- Convivente

D7 - Vive da sola? *
- Si
- No

D8 - Se no, con quante altre persone (oltre Lei)?
____

D9 - Titolo di studio *
- Licenza elementare
- Licenza media
- Diploma
- Laurea
- Post laurea

D10 - Indichi la Sua altezza *
Esprimere l'altezza in cm riportando solo il numero (esempio 165)
____

D11 - Indichi il Suo peso *
Indicare il peso in kg riportando solo il numero (esempio 60)
____

D12 - Attualmente ha una occupazione? *
- Sì, a tempo indeterminato
- Sì, a tempo determinato
- No, sono disoccupata
- No, sono una studentessa
- No, sono casalinga
- No, sono pensionata

D13 - Svolge attività fisica? *
- Sì, attività fisica moderata
- Sì, attività fisica intensa
- No, non svolgo attività fisica attualmente
- Non ho mai svolto attività fisica nella mia vita

D14 - Ha avuto una o più gravidanze? *
- Sì, in passato
- Sì, sono attualmente in attesa
- No
- Altro:___________________

D15 - Abitudine al fumo: attualmente Lei: *
- Fuma sigarette (tabacco)
- Fuma sigaretta elettronica
- No, ma ha fumato in passato
- No, non ha mai fumato

D16 - Le capita di bere una unità alcolica (1 bicchiere di birra o 1 bicchiere di vino o un superalcolico)? *

- Sì, solo durante i pasti (Passa alla domanda D16.1)
- Sì, prevalentemente durante i pasti (Passa alla domanda D16.1)
- Sì, prevalentemente fuori dai pasti (Passa alla domanda D16.1)
- Sì, solo fuori dai pasti (Passa alla domanda D16.1)
- Non bevo alcolici (Passa alla domanda D17)

D16.1 - In un giorno quante unità alcoliche (1 bicchiere di birra o 1 bicchiere di vino o un superalcolico) mediamente beve? *

____

D16.1.1 Quando le capita di bere, quante unità alcoliche (1 bicchiere di birra o 1 bicchiere di vino o un superalcolico) consuma nella stessa occasione?

____

Qualità dell'assistenza ricevuta

D17 - A che età ha saputo di avere l'endometriosi? *

____

D18 - Dopo quanti anni dalla comparsa dei sintomi ha avuto la diagnosi? (se è avvenuta dopo mesi indichi zero anni) *

- 0
- 1
- 2
- 3
- 4
- 5
- 6
- 7
- 8
- 9
- 10
- più di 10
- Non ho mai avuto sintomi

D19 - È mai stata visitata da centri/medici specializzati in endometriosi? *

- Si (Passa alla domanda D19.1)
- No (Passa alla domanda D20)
- Non ricordo/non so (Passa alla domanda D20).

<u>Centri/medici specializzati in endometriosi</u>

D19.1 - Questi centri/medici sono *

Seleziona tutte le voci applicabili.

- Pubblici
- Privati convenzionati
- Privati

D19.2 - Negli ultimi 12 mesi quante volte è stata visitata presso questi centri/medici? (Indichi un numero) *

Inserire in "Altro" il numero di volte, riportando solo il numero (esempio: 2) *(una sola risposta)*

- Mai (Passa alla domanda D20).
- Altro:______________________

<u>Visite negli ultimi 12 mesi in centri/medici specializzati</u>

D19.3 - In una di queste visite, è stata visitata in maniera approfondita, con ecografia e visita ginecologica? *

- ○ Sì
- ○ No
- ○ Non ricordo/non so

D19.4 - Se sì, quando è stata l'ultima volta che è stata visitata in tale maniera?

- ○ Negli ultimi 6 mesi
- ○ Più di 6 mesi fa
- ○ Non ricordo

D20 - Negli ultimi 12 mesi quante volte ha visto il Suo medico di famiglia per motivi legati all'endometriosi? *
Inserire in "Altro" il numero di volte, riportando solo il numero (esempio: 2) *(una sola risposta).*

- ○ Mai (Passa alla domanda D21).
- ○ Altro:__________________

D20.1 Quanto si ritiene soddisfatta dell'assistenza ricevuta dal Suo medico di famiglia da 0 a 5?
(0=molto insoddisfatta, 1=insoddisfatta, 2=discretamente soddisfatta, 4=soddisfatta, 5=molto soddisfatta) *
Risponda a questa domanda soltanto se negli ultimi 12 mesi si è recata dal Suo medico di famiglia. *(una sola risposta).*

- ○ 1
- ○ 2
- ○ 3
- ○ 4
- ○ 5

D21 - Negli ultimi 12 mesi è stata visitata da qualche specialista per problematiche correlate all'endometriosi? *

- ○ Sì (Passa alla domanda D21.1)
- ○ No (Passa alla domanda D22)
- ○ Non ricordo (Passa alla domanda D22)

D21.1 - Quale specialista l'ha visitata negli ultimi 12 mesi per problematiche correlate con l'endometriosi? *
Può scegliere una o più risposte

Seleziona tutte le voci applicabili.

- ☐ Endocrinologo
- ☐ Gastroenterologo
- ☐ Proctologo
- ☐ Urologo
- ☐ Radiologo
- ☐ Algologo (terapia del dolore)
- ☐ Fisiatra
- ☐ Fisioterapista
- ☐ Sessuologo
- ☐ Psicologo
- ☐ Nutrizionista
- ☐ Altro:_________________

D21.1.1 Aggiunga eventuali altri specialisti che l'hanno visitata negli ultimi 12 mesi per problematiche correlate all'endometriosi
____

D21.2 - Chi l'ha inviata dallo specialista? *
Può scegliere una o più risposte

Seleziona tutte le voci applicabili.

- ☐ Altro specialista

☐ Medico di famiglia
☐ Ha preso Lei l'iniziativa

D22 - Negli ultimi 12 mesi è stata ricoverata in ospedale per problematiche legate all'endometriosi, escluso il day hospital? *

o Sì
o No
o Non ricordo

<u>Vorremmo farLe ora qualche domanda sugli esami e farmaci che Le sono stati prescritti o consigliati dal Suo medico o dal centro per l'endometriosi e domande sugli interventi chirurgici.</u>

D23 - Attualmente, assume farmaci, prescritti o consigliati dal Suo ginecologo, per la terapia dell'endometriosi (quali pillola estroprogestinica, pillola progestinica, analoghi del GNRH)? *

o Sì
o No

D24 - Assume altri farmaci non prescritti o consigliati da un medico, per problematiche correlate all'endometriosi? *
Può selezionare più di una risposta

☐ Sì, antidolorifici
☐ Sì, integratori
☐ Sì, prodotti omeopatici
☐ No, assumo soltanto i farmaci prescritti o consigliati dal mio ginecologo
☐ No, non assumo alcun farmaco
☐ Altro:___________________

D25 - Presenta una o più delle seguenti complicanze, dovute all'endometriosi? *
Può selezionare una o più risposte. Aggiungere in "altro" ulteriori, eventuali, complicanze.

☐ Non presento complicanze
☐ Dolore pelvico cronico
☐ Dispareunia (dolore durante i rapporti)
☐ Problemi al pavimento pelvico
☐ Necessità di autocateterismi
☐ Neuropatia/problemi ai nervi
☐ Infertilità
☐ Isterectomia
☐ Salpingectomia
☐ Ovariectomia
☐ Stenosi intestinale
☐ Resezione intestinale
☐ Stomia intestinale/urostomia
☐ Resezione vescicale
☐ Sindrome aderenziale
☐ Altro:

D25.1 Aggiungere ulteriori, eventuali, complicanze
____

D26 - Ha mai subito un intervento chirurgico per endometriosi e/o per complicanze correlate all'endometriosi? *

☐ Sì (Passa alla domanda D26.1)
☐ No (Passa alla domanda D27)

D26.1 - Che tipo di intervento? *

Può barrare più risposte se ha subito più interventi.

☐ Laparoscopia diagnostica (per confermare o escludere la patologia)
☐ Laparoscopia operativa (per effettuare un trattamento. Esempio rimozione di cisti, lisi di aderenze, ecc)
☐ Laparotomia
☐ Non ricordo
☐ Altro:__________________________

Vorremmo chiederLe se ha ricevuto informazioni sui Suoi diritti come malata di endometriosi
D27 - È stata informata sulle agevolazioni cui ha diritto una paziente affetta da endometriosi (per le pazienti negli stadi clinici più avanzati cioè "moderato o III grado" e "grave o IV grado" è riconosciuto il diritto ad usufruire di un'esenzione per alcune prestazioni specialistiche di controllo)? *
--

- Sì (Passa alla domanda D27.1)
- No (Passa alla domanda D28)
- Non ricordo (Passa alla domanda D28).

D27.1 - Da chi ha ricevuto l'informazione? *
Può barrare più risposte

☐ Dal medico di famiglia
☐ Dal centro/medico specializzato in endometriosi
☐ Da associazioni di persone con endometriosi
☐ Da familiari, amici
☐ Da altre fonti (addetti del distretto o ASL, farmacisti, giornalisti, radio, televisione, pubblicità, ecc)
☐ Non ricordo

Le domande che seguono si riferiscono alla struttura che l'ha seguita più da vicino.
D28 - Negli ultimi 12 mesi, quale struttura ha seguito più da vicino la Sua malattia? *

- Centro specifico o medico specializzato in endometriosi (Passa alla domanda D28.1)
- Ambulatorio del medico di famiglia (Passa alla domanda D28.1)
- Nessuna struttura (Passa alla domanda D28.1a)
- Altro:_____________(Passa alla domanda D28.1)

Struttura che ha seguito più da vicino la Sua malattia
D28.1 - Come ritiene che siano gli orari della struttura? *

- Ottimi
- Adeguati
- Appena adeguati
- Non adeguati

D28.2 - Quanto sono accessibili i locali dove viene visitata, per esempio barriere architettoniche, mancanza di un ascensore, scalini, molto da camminare, ecc.? *

- Molto accessibili
- Abbastanza accessibili
- Accessibilità con una certa difficoltà
- Poco accessibili

D28.3 - Come giudica il livello di pulizia e gradevolezza? *

- Ottimo
- Buono
- Sufficiente
- Non sufficiente

D28.4 Come reputa le misure preventive anti-Covid applicate dalla struttura? *

- Ottime
- Buone
- Sufficienti
- Non sufficienti

D28.5 - Durante le Sue ultime visite, come Le sono sembrate la cortesia e la disponibilità di chi l'ha assistita? *

- Ottime
- Buone
- Soddisfacenti
- Non sufficienti

D28.6 - Durante le ultime visite quante volte Le sono state spiegate le cose in maniera a Lei comprensibile? *

- Sempre
- Spesso
- Qualche volta
- Mai

D28.7 - Durante le Sue ultime visite in ambulatorio, quante volte ha avuto l'impressione di essere ascoltata con attenzione? *

- Sempre
- Spesso
- Qualche volta
- Mai

<u>Vorrei farLe ora qualche domanda in particolare sull'ULTIMA visita nella struttura che l'ha seguita più da vicino</u>
D28.8 - Quanto tempo è trascorso dalla prenotazione alla visita? *

- Massimo un mese
- Tra uno e sei mesi
- Tra sei e 12 mesi
- Oltre un anno

D28.9 - Che tipo di visita era? *

- Nuova visita o visita di controllo, riprogrammata per problemi legati alla pandemia Covid
- Nuova visita
- Visita di controllo

D28.10 - L'ultima volta che è stata visitata, quanto tempo ha impiegato per raggiungere la struttura? *

- Meno di 15 minuti
- Tra 15 minuti e 30 minuti
- Tra 30 minuti e un'ora
- Più di un'ora

D28.11 - Quanto tempo ha dovuto attendere prima che si siano occupati di Lei? *

- Meno di 15 minuti
- Tra 15 e 30 minuti
- Tra 30 minuti e un'ora
- Più di un'ora

D28.12 - Quanto tempo è durato complessivamente il Suo ultimo controllo dall'arrivo fino all'uscita dalla struttura ambulatoriale? *
Specificare ore e/o minuti
____

D28.13 - Durante l'ultima visita, Le è stato dato un appuntamento ad una data precisa per la visita successiva? *

- Sì
- No, mi è stato detto di chiamare in seguito per fissare un appuntamento
- No, mi è stato detto che mi telefoneranno per fissarmi la data
- No, non mi è stato fissato un appuntamento
- Non ricordo

D28.14 - Globalmente come giudica il servizio che Le è stato offerto negli ultimi 12 mesi? *

-
- Ottimo
- Buono
- Sufficiente
- Non sufficiente

D28.15 - Come giudica il livello di coordinamento tra tutti i servizi e i diversi professionisti che si occupano della Sua malattia? *

- Ottimo
- Buono
- Sufficiente
- Non sufficiente

D28.16 - Qual è la fonte di informazioni che considera più utile per seguire e capire la Sua malattia? *

- Il centro per l'endometriosi o il mio ginecologo
- Il medico di famiglia
- Le associazioni di malati
- Interpreto da me stessa tutti i documenti
- Siti-web
- Social networks
- Non dispongo di una fonte di informazione veramente efficace

D28.17 - Ritiene che nell'ultimo anno in cui siamo stati colpiti dalla pandemia Covid, rispetto agli anni precedenti, ci siano stati cambiamenti nella gestione della sua malattia con riferimento ai seguenti aspetti? *

	**Aumentati**	**Diminuiti**	**Invariati**
Tempi di attesa dalla prenotazione alla visita			
Tempi impiegati per raggiungere la struttura			
Tempi di attesa prima della visita			

D28.18 - Ritiene che nell'ultimo anno, in cui siamo stati colpiti dalla pandemia Covid, rispetto agli anni precedenti, ci siano stati cambiamenti nella gestione della Sua malattia con riferimento ai seguenti aspetti? *

	**Migliorata/i**	**Peggiorata/i**	**Invariata/i**
Orari della struttura			
Accessibilità alla struttura			
Pulizia e gradevolezza			
Cortesia e disponibilità di chi l'ha assistita			
Comprensibilità delle spiegazioni			
Attenzione nell'ascolto			

D28.19 - Ritiene che i cambiamenti indicati possano essere attribuititi alla Covid? *

○ Sì
○ No

D28.20 - Se no, a cosa?
____

Se negli ultimi 12 mesi non è stata seguita da alcuna struttura

D28.1a - Quanti mesi sono trascorsi dall'ultima visita? *
____

D28.2a - Perchè negli ultimi 12 mesi non si è fatta seguire da una struttura? *

☐ Per scelta personale legata alla paura del contagio Covid
☐ Perché impossibilitata per motivi di salute legati alla Covid
☐ Ha già un appuntamento per una visita
☐ È stata seguita in telemedicina
☐ La visita è stata annullata/spostata per problematiche legate alla pandemia
☐ Altro:__________________________

D29 - In rapporto a tutti gli argomenti di cui abbiamo discusso, che cosa proporrebbe per migliorare l'assistenza delle donne malate di endometriosi della Sua Regione?
____
____

D30 - Soffre di altre patologie, oltre all'endometriosi? *

○ Sì
○ No

D31 - Se ha risposto sì, se vuole può indicare quali
____

D32 - È vaccinata per la Covid-19? *

○ Sì
○ No, non ancora
○ No, non intende vaccinarsi

D33 - Come ha trovato questo questionario? *

○ Gruppo privato su Social Network
○ Gruppo pubblico su Social Network
○ Post sulla bacheca di un contatto Facebook
○ Instagram
○ Altro Social Network
○ Servizi di messagistica istantanea (WhatsApp, Telegram, Messanger, ecc)
○ Associazione donne con endometriosi
○ Email
○ Altro _________________

STATO DI SALUTE SF36

Il questionario è concluso può cliccare su "invio". La ringraziamo per la collaborazione
